# NFATc4 Knockout Promotes Neuroprotection and Retinal Ganglion Cell Regeneration After Optic Nerve Injury

**DOI:** 10.1007/s12035-024-04129-0

**Published:** 2024-04-19

**Authors:** Joanna Mackiewicz, Julia Tomczak, Malwina Lisek, Agata Sakowicz, Feng Guo, Tomasz Boczek

**Affiliations:** 1 Department of Molecular Neurochemistry, Medical University of Lodz, Lodz, Poland; 2 Department of Medical Biotechnology, Medical University of Lodz, Lodz, Poland; 3 Department of Pharmaceutical Toxicology, China Medical University, Shenyang, China

**Keywords:** Optic nerve injury, NFATc4 transcription factor, Retinal ganglion cell survival, Intravitreal gene delivery, Apoptotic gene expression

## Abstract

Retinal ganglion cells (RGCs), neurons transmitting visual information via the optic nerve, fail to regenerate their axons after injury. The progressive loss of RGC function underlies the pathophysiology of glaucoma and other optic neuropathies, often leading to irreversible blindness. Therefore, there is an urgent need to identify the regulators of RGC survival and the regenerative program. In this study, we investigated the role of the family of transcription factors known as nuclear factor of activated T cells (NFAT), which are expressed in the retina; however, their role in RGC survival after injury is unknown. Using the optic nerve crush (ONC) model, widely employed to study optic neuropathies and central nervous system axon injury, we found that NFATc4 is specifically but transiently up-regulated in response to mechanical injury. In the injured retina, NFATc4 immunolocalized primarily to the ganglionic cell layer. Utilizing NFATc4^−/−^ and NFATc3^−/−^ mice, we demonstrated that NFATc4, but not NFATc3, knockout increased RGC survival, improved retina function, and delayed axonal degeneration. Microarray screening data, along with decreased immunostaining of cleaved caspase-3, revealed that NFATc4 knockout was protective against ONC-induced degeneration by suppressing pro-apoptotic signaling. Finally, we used lentiviral-mediated NFATc4 delivery to the retina of NFATc4^−/−^ mice and reversed the pro-survival effect of NFATc4 knockout, conclusively linking the enhanced survival of injured RGCs to NFATc4-dependent mechanisms. In summary, this study is the first to demonstrate that NFATc4 knockout may confer transient RGC neuroprotection and decelerate axonal degeneration after injury, providing a potent therapeutic strategy for optic neuropathies.

**Supplementary Information:**

The online version contains supplementary material available at 10.1007/s12035-024-04129-0.

## Introduction

Retinal ganglion cells (RGCs), a highly specialized type of neurons, transmit visual information from the retina to the visual processing centers of the brain. Because the unidirectional optic nerve is formed exclusively by axons projecting by RGCs, it is highly vulnerable to various injuries, leading to irreversible loss of RGC function. The progressive death of RGCs is a crucial element in the pathophysiology of glaucoma, characterized by the progressive degeneration of the optic nerve and resulting in irreversible blindness. Over the decades, numerous studies have investigated the mechanisms underlying RGC death and identified several neuronal abnormalities associated with antioxidant imbalance [1], neuroinflammation [2], mitochondrial metabolism alterations [3], ischemia/hypoxia [4], or vascular deficits [5]. Recent studies have also demonstrated a promising effect of small molecules and virus-based gene therapies in pre-clinical models of glaucoma [6–9].

Considerable effort is currently dedicated to unraveling molecular changes underlying disease progression and understanding signaling pathways that can be manipulated to enhance RGC survival. The most effective approaches involve the exogenous administration of neurotrophic factors or apoptotic pathway inhibitors, such as brain-derived neurotrophic factor (BDNF), ciliary neurotrophic factor (CNTF), neurotrophin-4, or their combination with oncomodulin or osteopontin [10–16]. In the majority of cases, the effects are only transitory, even with long-lasting treatments. Similarly, caspase inhibitors provide only transient neuroprotection [17–23]. Strategies based on the knockdown or overexpression of other prominent regulators of RGC growth and survival, such as Elk-1 [24], Sigma-1R (σ-1r) [25], MEF2 [26], or PTEN [27, 28] have also been tested. Despite the tremendous progress in several molecular interventions targeting RGC survival following injury, most of them present only limited effectiveness in clinical interventions. This limitation likely arises from the choice of inappropriate target(s) or time window. Therefore, the elucidation of mechanisms leading to RGC death is essential to target the right signaling molecule or decipher the therapeutic time-window.

Originally described as important regulators of immune function [29], NFATs are now implicated in the regulation of neuronal morphogenesis, plasticity, and the response to neurotrophin and electrical stimulation [30–32]. In humans, the NFAT family comprises five transcription factors named NFAT1 (NFATc2), NFAT2 (NFATc1), NFAT3 (NFATc4), NFAT4 (NFATc3), and NFAT5, which, all but the last one, are regulated by Ca^2+^ - activated protein phosphatase-2B/calcineurin (CaN). The nature, source and timing of upstream signals regulating Ca^2+^/CaN activity and the flexibility of NFAT in cooperating with other transcriptional activators or repressors make an important contribution to neuronal response to external stimuli. This relationship is seen in both developing and mature neurons. For instance, profound defects in axon projections were observed in embryos with combined deletion of either NFATc3 or NFATc4 (c3/c4 mutants) or NFATc2, NFATc3, and NFATc4 (c2/c3/c4 mutants) [33].These defects were reproduced by in utero administration of cyclosporine A (CsA) – a potent inhibitor of CaN. CaN and NFATs are also essential for neurotrophin-induced neuronal outgrowth and survival [34].

The role of NFATs in RGCs is not yet fully understood, as it becomes increasingly apparent that these cells can selectively activate specific NFAT isoforms. Recently, NFATc2 and NFATc3 have been identified as the major isoforms expressed in retina [35]. NFATc4, present in low amounts, was significantly upregulated in RGCs following light-induced damage and was associated with increased neuronal apoptosis [36]. Despite the obvious role of NFAT in neuronal function, the contribution of NFAT isoforms to retinal degeneration following pro-death insults has not been widely studied. This is of paramount importance as the progressive loss of RGCs is a hallmark common to the majority of optic neuropathies, including glaucoma, often leading to permanent blindness [37–40]. Traumatic optic neuropathy and glaucomatous injury can be mimicked by mechanical optic nerve crush (ONC), which serves as a preclinical model of neuronal survival and regeneration, as it similarly induces RGC death and degeneration. In this model, the lesion severs all of the RGCs’ axons, ensuring high reproducibility and precise control of the injury site [41–45]. As optic neuropathy can be linked with other CNS diseases, this model can also be used in CNS degeneration studies to unravel degenerative mechanisms and test neuroprotective therapies. Using ONC, we provide evidence for the critical role of NFATc4 in RGC survival following injury.

## Materials and Methods

### Animals

All animal procedures were carried out according to the Association for Research in Vision and Ophthalmology (ARVO) guidelines for the use of animals in ophthalmic and vision research. The experimental protocols were approved by the Institutional Animal Care and Use Committee at the Medical University of Lodz. All mice used in this project were C57BL/6. The animals were group-housed in laboratory cages and kept under a controlled temperature (23 ± 2 °C) with a 12-h light/dark cycle and with food and water provided ad libitum. Nfatc4^−/−^ mice (B6;129 S-*Nfatc4*^tm1Grc^/J, strain #027581) and Nfatc3^−/−^ mice (B6;129S2-*Nfatc3*^tm1Glm^/J, strain #010589) were obtained from the Jackson Laboratory (USA). Both males and females were used in all experimental procedures.

### Lentiviruses

Lentiviruses were employed for in vivo delivery of NFATc4-GFP to NFATc4^−/−^ mouse retina, owing to the limited capacity of adeno-associated virus serotype 2. Lenti ORF particles, Nfatc4 (GFP-tagged) transcript variant 2 (reference sequence NM_001168346.1, 4036 bp), as well as Lenti ORF control particles of pLenti-C-mGFP-P2A-Puro, were generated by Origene (USA). NFAT luciferase reporter lentivirus and firefly luciferase lentivirus were sourced from BPS Bioscience (USA). Lenti-VIVIT-GFP was produced with pLV-VIVIT-GFP plasmid (Addgene#188,707) using Lenti-X Packaging Single Shots technology from Takara Bio (USA). All viral titers were > 10^7^ TU/ml. In vitro or in vivo transduction was caried out as specified for each experiment.

### Isolation of Primary Neurons

RGCs were purified from postnatal (P8-P10) mouse pups using a procedure essentially described in [46] with some modifications. Isolated retinas were washed 3 times with Dulbecco Phosphate Buffer Saline (DPBS) and digested with papain (16.5 U/ml, VWR, USA) for 30 min at 37^o^C. After trituration, papain activity was inhibited by adding ovomucoid solution (1.5 mg/ml, Merck, USA), and the cell suspension was centrifuged at 250 x g for 10 min. The resulting pellet was mixed with DPBS supplemented with 5 µg/ml insulin (Merck, USA) and transferred to anti-macrophage antibody-coated petri dishes for a 45 min incubation. Suspended cells were subsequently transferred to petri dishes containing anti-Thy 1.2 antibody conditioned media and further incubated for 45 min to isolate RGCs. Petri dishes were washed multiple times, and RGCs were released by trypsinization. RGCs were routinely seeded at the density of 50.000 cells/well in a 24 well plate coated with poly-D-lysine (10 µg/ml) and laminin (1 µg/ml). Cells were cultured in serum-free media supplemented with sodium pyruvate (1 mM), B27 (1:50, Thermo Fisher, USA), BDNF (50 ng/ml, Thermo Fisher, USA), CNTF (10 ng/ml, VWR, USA), forskolin (5 mM, Merck, USA), insulin (5 µg/ml, Merck, USA), N-acetyl cysteine (5 µg/ml, Merck, USA), L-glutamine (1 mM, VWR, USA) and triiodothyronine (40 ng/ml, Merck, USA), unless otherwise specified, at 37^o^C/5% CO_2_ and in a humidified atmosphere. The purity of isolated RGCs was verified by staining with antibodies recognizing RNA binding protein with multiple splicing (RBPMS, 1:1000, Aves Labs, USA) and typically exceeded 95%.

Primary hippocampal neurons were prepared following the methodology outlined in our previous publication [47]. In brief, hippocampal cultures were derived from Sprague-Dawley E18 embryos. Hemispheres were dissected in HBSS buffer on ice, trypsinized for 30 min at 37^0^C, centrifuged at 250 x g for 2 min, and then triturated with a fire-polished glass pipette. The dissociated neurons were seeded on nitric acid-soaked glass coverslips coated with poly-L-lysine in plating medium (10% v/v horse serum in DMEM). After 4 h, the medium was replaced with Neurobasal maintenance medium supplemented with 2% B27, 1 mM glutamine, 1 mM sodium pyruvate, and 5 µg/ml insulin. For cultures lasting beyond 4 days, half of the medium was removed on day 3 or 4 and replaced with an equal volume of fresh medium.

### In Vitro Survival and Axon Elongation Assay

Isolated RGCs (~ 200,000 cells) were promptly electroporated with NFATc4 ON-TARGETplus siRNA oligonucleotides or ON-TARGETplus scrambled siRNA, both administered at 1 nmol per electroporation (Horizon Discovery, USA). The electroporation was conducted according to the method detailed elsewhere [48]. Subsequently, cells were cultured for up to 3 days, and double-stained for annexin V-FITC (Abcam, UK) for 10 min in growth media to label apoptotic cells and Calcein Red™ (Thermo Fisher, USA) for 20 min to label live cells. Images of 10–12 randomly selected fields were captured in growth media at 2 and 72 h using a Leica DMi8 inverted microscope to quantify cell survival. The survival was quantified using ImageJ and was normalized to scrambled siRNA-treated RGCs.

For the axon elongation assay, isolated RGCs were electroporated and seeded at a low density of ~ 5000–6000 cells/well in a 48 well plate, followed by a 3-day culture period. RGCs were then fixed with 3.8% paraformaldehyde (PFA) and permeabilized with 0.2% Triton X-100 in PBS. After several washes with PBS, cells were labelled with anti-βIII tubulin (1:500, Cell Signaling, USA) overnight at 4^0^C. The plates were next probed with secondary antibodies conjugated to Alexa Fluor 488 (1:500, Thermo Fisher, USA) for 6 h at room temperature. Nuclei were counterstained with DAPI (VWR, USA) at a dilution of 1:5000. Images were acquired on a Leica DMi8 inverted microscope, and the longest neurite per cell (∼ 20 cells on average in each experiment) was measured using the ImageJ Neurite Tracker tool. The results were normalized to the average axonal length in scrambled siRNA-treated RGCs.

### Optic Nerve Crush (ONC) and Intravitreal Injections

For optic nerve crush, C57BL/6 male and female mice at the age 7–8 weeks were randomly assigned to the treatment group. The crush procedure followed protocols essentially described in previous studies [28, 49] with minor modifications. Mice were anesthetized with 20 mg/kg IP xylazine (BioWet, Poland) and 100 mg/kg IP ketamine (BioWet, Poland). A drop of 0.5% proparacaine was applied to the eye. The optic nerves were exposed from the outer canthus behind the globe, and the crush was performed ∼ 2 mm behind the eyeball for 3 s using extra-fine forceps. The contralateral control eye underwent the same procedure but was spared from the crush. Care was taken to avoid damaging blood vessels in the retina. Post-operative analgesia was provided with 0.5 mg/ml buprenorphine (Bupaq Multidose, Orion Pharma, Poland). In lentivirus transduction experiments, mice under isoflurane anesthesia were intravitreally injected through the sclera with 2 µl of lentivirus in PBS using a 31-gauge needle (Hamilton) connected to a 5 µL Hamilton syringe. Care was taken to avoid damage to the lens. Viral injections were performed approximately 2 weeks before ONC to allow for sufficient gene expression. Animals experiencing any postoperative complications such as excessive bleeding or swelling, retinal ischemia or cataract were excluded from the cohort at any time after procedure.

### Retinal Flat-Mount and RGC Count

The retinal flat-mount preparation procedure was performed on mice deeply anesthetized with isoflurane, followed by intraperitoneal administration of ketamine/xylazine. Subsequently, the mice were sacrificed through transcardial perfusion with 4% paraformaldehyde (PFA). The methodology for retinal flat mount was adapted from [50] and used in our previous study [47]. In brief, the eyes were removed, post-fixed with 4% PFA for 2 h at room temperature, and the retinas were dissected. After several washes with PBS, retinas were permeabilized with Triton X-100 and incubated overnight at 4^0^C with a primary anti-RBPMS antibody (1:500, Aves Labs, USA) in a blocking buffer (PBS with 10% goat serum). Following washing with PBS, retinas were incubated with secondary antibodies conjugated to Alexa Fluor 488 (1:500,Thermo Fisher, USA) for 2 h at room temperature. Subsequently, retinas were flat-mounted in H-1000 mounting medium (VectorLabs, USA) on glass slides. Scan images were acquired with Leica SP8 confocal laser scanning microscope. The counting of RBPMS-positive cells was carried out in a manner described in a previous publication [47]. This process, performed by an experienced researcher in a masked fashion, involved assessing RGC cell density per mm^2^ or percentage change relative to the sham-operated contralateral eye or ONC-treated wild-type.

### Anterograde Labelling, Quantification of Regeneration and Axon Degeneration Analysis

Two days prior to optic nerve harvesting, 2 µl of cholera toxin subunit B (CTB, 2 µg/µl, Thermo Fisher, USA) were intravitreally injected to visualize axons and nerve terminals of surviving RGCs. Animals were perfused with 4% PFA before the collection of optic nerves. The optic nerves were cryopreserved overnight in 30% sucrose at 4^0^C and then mounted in Optimal Cutting Temperature mounting medium (Thermo Fisher, USA). Longitudinal sections, 10 μm thick, were cut for optic nerves and imaged using a DMi8 fluorescence microscope (Leica, Germany). The sections were analyzed as described previously [41]. The number of CTB-positive axons passing 0.1, 0.25, 0.5, 0.75, 1.0, 1.25 mm from the crush site was manually counted. The total number of CTB-positive axons per optic nerve was calculated using methods outlined in a previous study [51]. This approach provides a quantitative assessment of axon survival and regeneration in response to experimental conditions.

Axonal integrity was assessed one week after ONC through βIII-tubulin staining. This technique enables the measurement of protein abundance within axons [52] and has recently been employed to demonstrate delayed optic nerve degeneration in response to pharmacological inhibition of aldolase reductase [53]. For immunostaining, 10-µm-thick cryosections were probed with anti-βIII-tubulin antibodies (1:500, Cell Signaling, USA), followed by incubation with secondary antibodies conjugated to Alexa Fluor 594 (1:1000, Thermo Fisher, USA). The density of βIII-tubulin was measured in a 500 × 200 μm area immediately after the crush site [54], following the protocol described in [53]. Optic nerves were imaged at the same intensity using a Leica DMi8 fluorescence microscope with a 10x objective. This approach provides insights into the preservation or alterations in βIII-tubulin expression, reflecting axonal structural integrity following the ONC procedure.

### Western Blotting

Retinas were isolated and lysed using RIPA buffer supplemented with a protease and phosphatase inhibitor cocktail. The total protein content was quantified colorimetrically with the Bio-Rad Protein Kit Assay (Bio-Rad, USA). Subsequently, 10–30 µg of the protein samples were separated in 4–20% gradient polyacrylamide gels and transferred to a nitrocellulose membrane using a semi-dry method. The membranes were blocked with 10% goat serum in TBST-T buffer (10 mM Tris-HCl, pH 7.4, 150 mM NaCl, and 0.05% Tween-20) for 2 h at room temperature. Next, the membrane was incubated with primary antibodies recognizing NFATc4 (1:750,, Abcam, UK), caspase-3 (1:1000,, Thermo Fisher, USA), or GAPDH (1:3000,, Merck, USA) for 24 h at 4^o^C. Following three washes in TBS-T, the membrane was probed with secondary antibodies (1:5000, Merck, USA) conjugated to horse radish peroxidase for 2 h at room temperature. ECL western blot system (Bio-Rad, USA) was used to visualize immunoreactive bands. The membranes were scanned densitometrically, and the optical density of bands was quantified using ImageJ. The results are expressed as arbitrary units after normalization to the endogenous GAPDH level, providing a quantitative assessment of protein expression levels.

### Total RNA Isolation, Real-Time PCR and Microarray Screening

Total RNA was extracted from the retina using Trizol reagent (Thermo Fisher, USA) following the manufacturer’s protocol. Single-stranded cDNA was synthesized from 1 µg of isolated RNA using M-MLV reverse transcriptase (Promega, USA) with oligo(dT) primers. Real-time PCR reactions were carried out under the following conditions: an initial denaturation at 95 °C for 15 min, followed by 40 cycles at 95 °C for 15 s, 60 °C for 30 s, and 72 °C for 30 s, using the Abi Prism 7000 sequence detection system using Eva Green Master Mix. Primers used in the reactions: Nfatc1 (NM_198429), Nfatc2 (NM_010899), Nfatc3 (NM_010901), Nfatc4 (NM_023699), Gapdh (NM_008084) were purchased from Origine (Germany). The specificity of the PCR product was assessed by running a melting curve. The relative expression of the gene was determined using the ΔCt method [55], with endogenous Gapdh expression used for data normalization.

For microarray screening, cDNA amplified from 2 µg of retinal RNA was hybridized with RT² Profiler™ PCR Array Mouse Apoptosis (Qiagen, USA), and the reaction was performed using HOT FIREPol® EvaGreen® qPCR Mix Plus (Solis Biodyne, Estonia). The real-time PCR conditions included an initial cycle at 95 °C for 10 min, followed by cycles at 95 °C for 15 s, 60 °C for 1 min, and a dissociation curve at 95 °C for 1 min, 55 °C for 30 s, and 95 °C for 30 s. The fold change was calculated by a method of Livak and Schmittgen [55]. Data were analyzed using Qiagen PCR Array Data Analysis Web Portal. The microarray analysis was run in triplicate, and the RT^2^ software averaged the triplicate normalized expression for each gene (ΔCt) before calculating ΔΔCt between the control (WT after ONC) and experimental group (NFATc4^−/−^ after ONC). Housekeeping genes used for normalization were selected based on the recommendations of Vandesompele et al. [56]. Two Microarray Quality Control studies demonstrated that a P-value calculation based on fold change could be considered sufficient for obtaining reproducible results across microarray analyses, including RT^2^ Profiler PCR Arrays [57, 58].

### Retina Cryosection Staining

One or five days after ONC, eyes were removed, incised at the cornea for better penetration, and immersed in a 3.8% PFA solution for 48 h at 4^o^C. Subsequently, the eyes were incubated in a 30% sucrose solution for an additional 4 h, embedded in OCT medium, and cryosectioned into 10-µm thickness. Retinal sections were blocked with 5% bovine serum albumin (BSA)/0.3% Triton X-100 in PBS for 10 min at room temperature. Primary antibodies against NFATc4 (1:500, Merck, USA), RBPMS (1:500,, Aves Labs, USA), or cleaved caspase-3 (1:200,, Cell Signaling, USA) were applied in BSA-containing blocking buffer for 1 h at room temperature. Following several washes with PBS, sections were stained with Alexa Fluor 488-conjugated secondary antibodies (1:500, Thermo Fisher, USA) for 1 h before final washing and mounting. Images were acquired with a Leica SP8 confocal laser scanning microscope. The nuclei of retinal cells were counterstained with DAPI (1:5000, VWR, USA). For quantitative analysis, caspase-3 positive cells were counted in RGC layer of the retina using ImageJ counting plugin 1.41 software. The density profiles were expressed as the mean number of caspase-3 positive cells per mm2, providing a quantitative assessment of apoptotic cell density in the RGC layer.

### In Vitro Luciferase Reporter Assay

NFAT transcriptional activity was assessed following a protocol similar to [59] with some modifications. In brief, lentiviral particles were designed to carry a firefly luciferase gene under the control of the NFAT response element positioned upstream of the minimal TATA promoter. Primary hippocampal neurons were transduced with Lenti-NFAT luciferase reporter and Lenti-luciferase at DIV0, and the neurons were cultured for 3 days. NFAT transcriptional activity in control cells, NFATc4-overexpressing cells, or VIVIT-expressing cells was measured in cell lysates using the Dual-Glo Luciferase Assay System (Promega, USA) according to the manufacturer’s instructions. The expression of the NFAT luciferase reporter was normalized to the expression of firefly luciferase. The fold increase of normalized NFAT luciferase reporter was then calculated over the baseline values.

### Electroretinography (ERG)

ERG was conducted following a protocol similar to [60] with some modifications. Mice were dark-adapted overnight and then anesthetized with intraperitoneal administration of ketamine/xylazine (100 mg/kg; 20 mg/kg, BioWet, Poland). Both eyes were treated with 1% atropine sulfate, 2.5% phenylephrine hydrochloride, and 0.5% proparacaine hydrochloride (all from Thermo Fisher, USA) for approximately 2 min. Electrodes were carefully positioned onto the corneas of both eyes using hypromellose ophthalmic solution. Single flashes of 10 ms duration with an intensity of 2.48 cd-s/m^2^ were applied for stimulation under scotopic conditions. The recordings were performed using the UTAS-E2000 (Universal Testing and Analysis System Electrophysiologic 2000) equipment (LKC Technologies, USA). This method allows for the assessment of retinal function through the measurement of electrical responses to light stimuli, providing valuable information on the integrity and activity of the retina.

### Statistics

Statistical analysis was performed using GraphPad Prism 8.0.1 version. The normality of data was checked with Shapiro-Wilk test. Statistical significance was determined using Student’s t-test, one- or two-way ANOVA with multiple comparison post hoc correction.

## Results

### NFATc4 Is Transiently Increased After Optic Nerve Injury

**Fig. 1:**
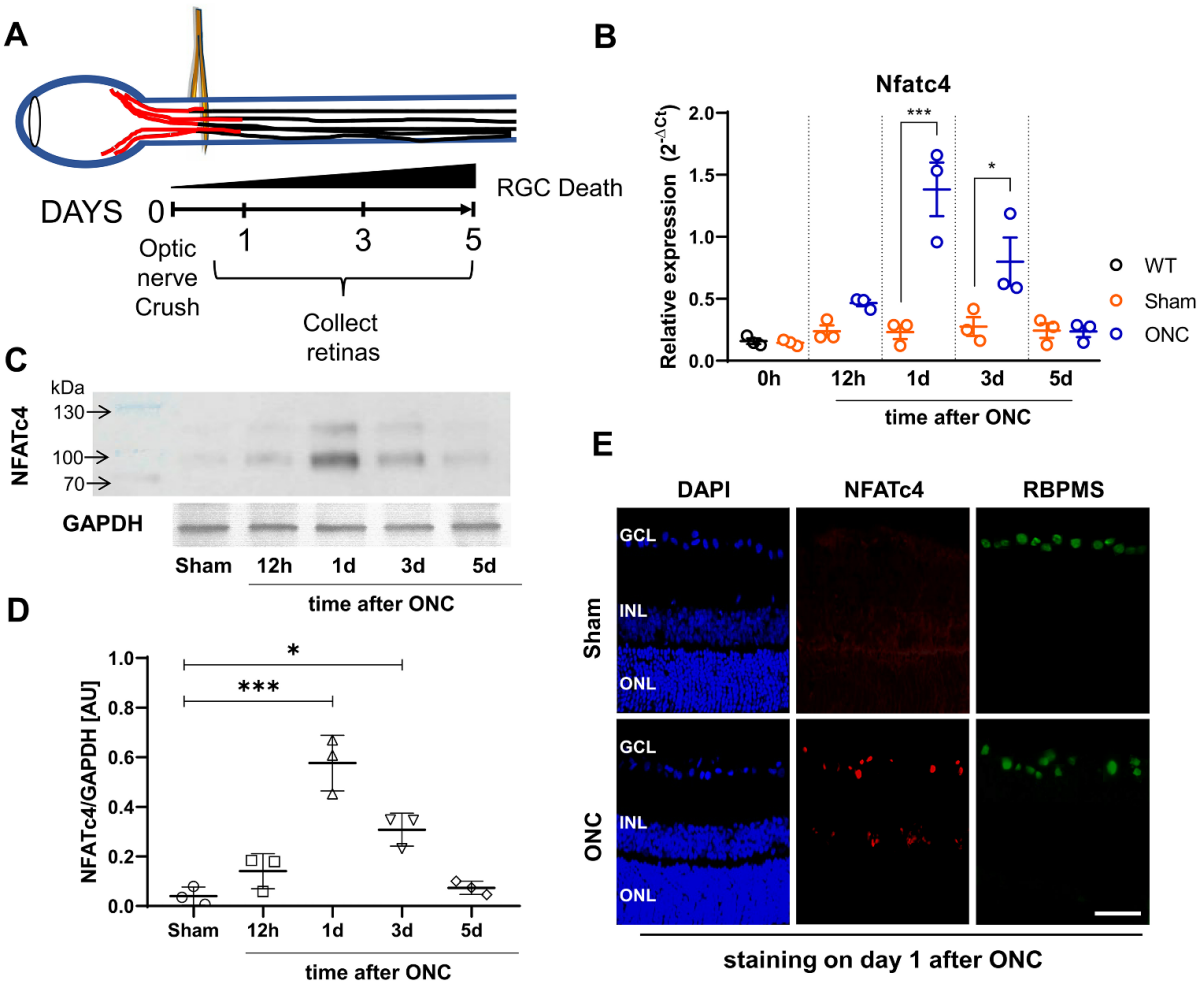
The changes in NFATc4 in the adult retina following optic nerve crush (ONC). (A) Experimental design scheme. Retinas were collected at different time points (either 12 h, 1, 3 or 5 days) after ONC for subsequent experiments. (**B**) Changes in NFATc4 mRNA expression level measured by real-time PCR. Raw data were normalized to Gapdh expression, and the relative fold change was calculated using 2^−ΔCt^ method, *n* = 3 at different time points after ONC. (**C**) Representative western blot for NFATc4 and quantification of protein expression after normalization to GAPDH protein level, *n* = 3 at different time points after ONC. Data are presented as means ± SEM with individual values indicated on graphs. * *P* < 0.05, ** *P* < 0.01, *** *P* < 0.001. (**D**) Quantification of NFATc4 protein level in Sham-operated or ONC retinas following normalization to endogenous GAPDH level, *n* = 3. AU – arbitrary units. (**E**) NFATc4 expression in sectioned adult retina following optic nerve injury. Representative micrographs of retina sections were evaluated for NFATc4 expression on day 1 after optic nerve crush. RBPMS was stained to visualize RGCs, and DAPI was used to locate ganglion cell layer (GCL), inner nuclear layer (INL), and outer nuclear layer (ONL). Strong immunoreactivity was present within the GCL. Scale bar: 50 μm

The expression of NFAT isoforms has been previously established in an intact mouse retina [35]. At the mRNA level, NFATc3 was identified as the predominant isoform, although the expression of NFATc2 was also readily detected. In contrast, the expression of NFATc1 and NFATc4 was relatively low [35]. To investigate the potential involvement of the NFAT transcription factor family in RGC survival and regeneration following injury, we initially assessed the changes in NFATc1-c4 expression following ONC. This model was chosen due to the predictable and consistent pattern of RGC death after optic nerve lesion, facilitating the tracking of molecular events underlying RGC loss [61]. The expression of NFATc4 increased significantly following ONC, peaking on day 1 and returning to baseline levels on day 5 when compared to contralateral control and GAPDH (Fig. 1A-D). This suggests that NFATc4 may be involved in RGC response to injury.

NFATc4 staining conducted on day 1 post-crush revealed a concentration of the signal within the GCL of the retina (Fig. 1E). We also observed a more muted expression of NFATc4 within the inner nuclear layer, which could contribute to the changes detected by Western blot. However, the alterations identified after optic nerve crush are likely attributed to responses from RGCs, as optic nerve injury is a well-characterized model of RGC degeneration [62]. We additionally examined the expression of NFATc1-c3, but no significant changes at the mRNA level were detected after ONC (see Supplementary Fig. 1).

### NFATc4 Is Important for RGC Growth and Survival in Vitro

In an initial exploration of NFATc4’s significance in optic nerve degeneration, purified RGCs were cultured, and NFATc4 level was selectively reduced using siRNA. Given previous findings indicating the pro-survival effects of neurotrophins and elevated cAMP level, RGCs were initially cultured in the presence of forskolin (an adenylyl cyclase activator), BDNF, and CNTF. It is noteworthy that in vitro culturing and electroporation of RGCs can induce ongoing cell death even in a rich maintenance media, partially mimicking conditions during in vivo optic nerve injury.

**Fig. 2:**
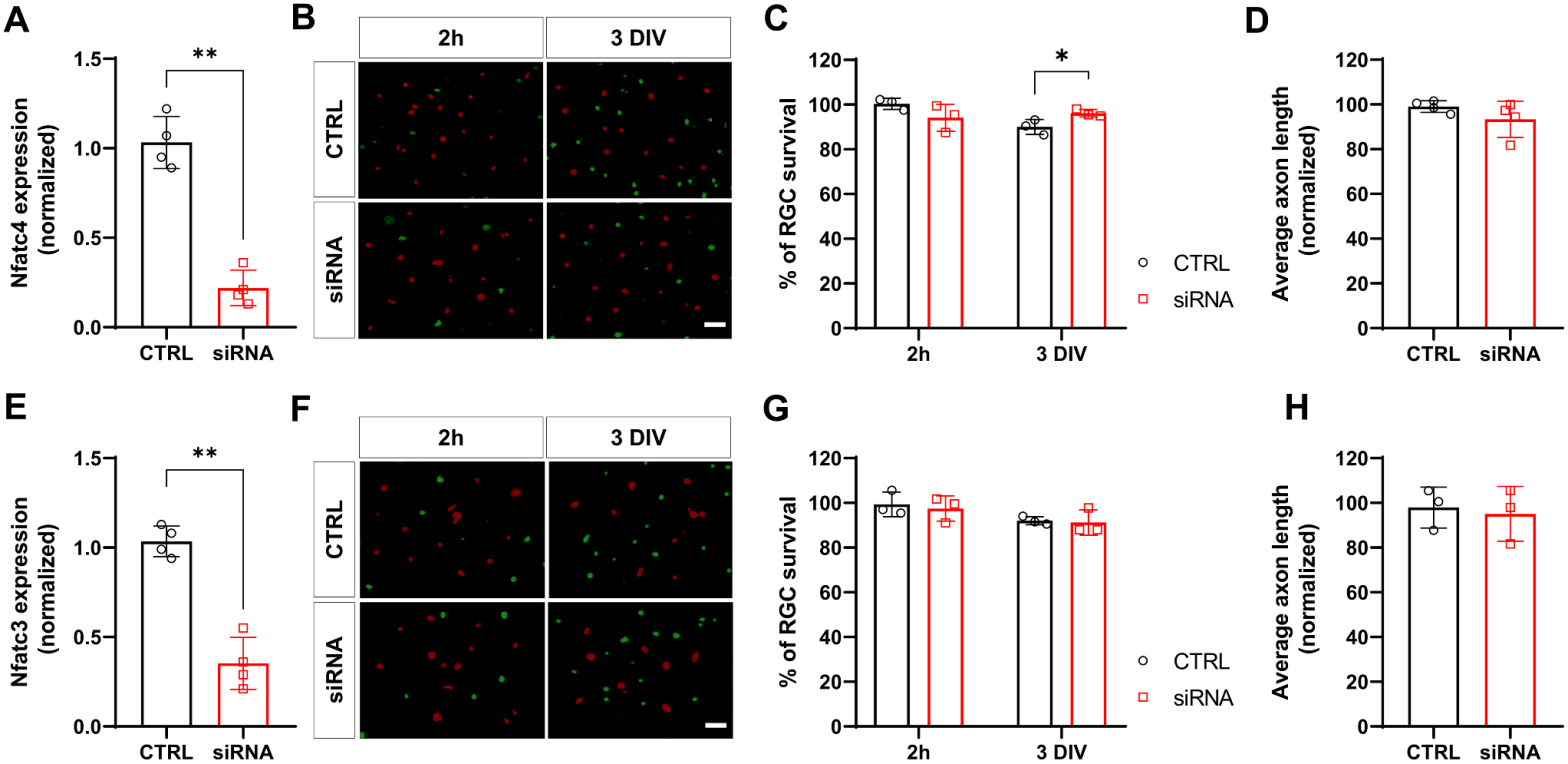
NFATc4 in RGC survival and axon elongation in vitro. (**A**) The efficiency of Nfatc4 silencing calculated using 2^−ΔΔCt^ method relative to scrambled siRNA control. The data were normalized to the endogenous Gapdh expression level, *n* = 4. (**B**) Purified RGCs were electroporated with either NFATc4 siRNA or control and stained with Annexin V-FITC and Calcein Red to visualize apoptotic (green) and live (red) cells, respectively, at different time points. Scale bar: 100 μm. (**C**) RGC survival normalized to scrambled siRNA-treated RGCs for indicated time points. n = 3. (**D**) Average axon length of the Nfatc4 siRNA group at DIV3 normalized to control RGCs. n = 3. (**E**) The efficiency of Nfatc3 silencing calculated with 2^−ΔΔCt^ method after normalization to Gapdh expression. The expression level in scrambled siRNA-transfected cells was taken as 1. n = 4. (**F**) Purified RGCs electroporated with either control or NFATc3 siRNA stained with Annexin V-FITC (green) and Calcein Red (red). Scale bar: 100 μm. (**G**) RGC survival following NFATc3 siRNA treatment normalized to scrambled siRNA control for respective time points. n = 3. (**H**) Average axon length of Nfatc3 siRNA-treated cells at DIV3 normalized to control RGCs. n = 3. The data are presented as means ± SEM. * P < 0.05, ** P < 0.01

Two days post-electroporation, NFATc4 expression was suppressed by nearly 80% relative to the control siRNA group (Fig. 2A). NFATc4 silencing slightly increased RGC viability but did not impact axonelongation, suggesting a role for NFATc4 in neuronal survival (Fig. 2B-D).

To confirm the specificity of the observed change, we subsequently investigated whether a similar effect could be achieved by silencing NFATc3, the main NFAT isoform expressed in the retina (Fig. 2.). In contrast to NFATc4, silencing NFATc3 with an efficiency of approximately 70% (Fig. 2E) did not exert a pro-survival effect (Fig. 2F-H). Control experiments performed 2 h following electroporation showed no differences between NFATc4, NFATc3 and control siRNA groups, indicating that the observed effects were not due to differential electroporation.

### Nfatc4^−/−^ Mouse has Higher Baseline RGC Survival After Injury

While the conditions of RGC culturing are termed “stressed”, it is essential to note that there are differences in the underlying mechanisms between RGC growth and survival in vitro and those governing RGC survival in vivo. Therefore, the observed significance of NFATc4 to RGC survival in vitro may not necessarily be replicated in vivo during optic nerve injury. To explore this hypothesis, we utilized a mutant mouse with genetically ablated Nfatc4 (Nfatc4^−/−^ mouse). For rescue experiments, we delivered GFP-tagged NFATc4 to the retina using lentiviral vectors as the size of NFATc4 exceeds the capacity of adeno-associated virus, serotype 2 (AAV2). The transgene delivered via lentiviral-mediated transfer has been demonstrated to be expressed preferentially in retinal neurons of GCL and a small population of retinal pigment epithelial cells and lasts at least, up to 3 weeks [63]. Nfatc3^−/−^ mouse was included in the study to determine the specificity of observed changes.

**Fig. 3:**
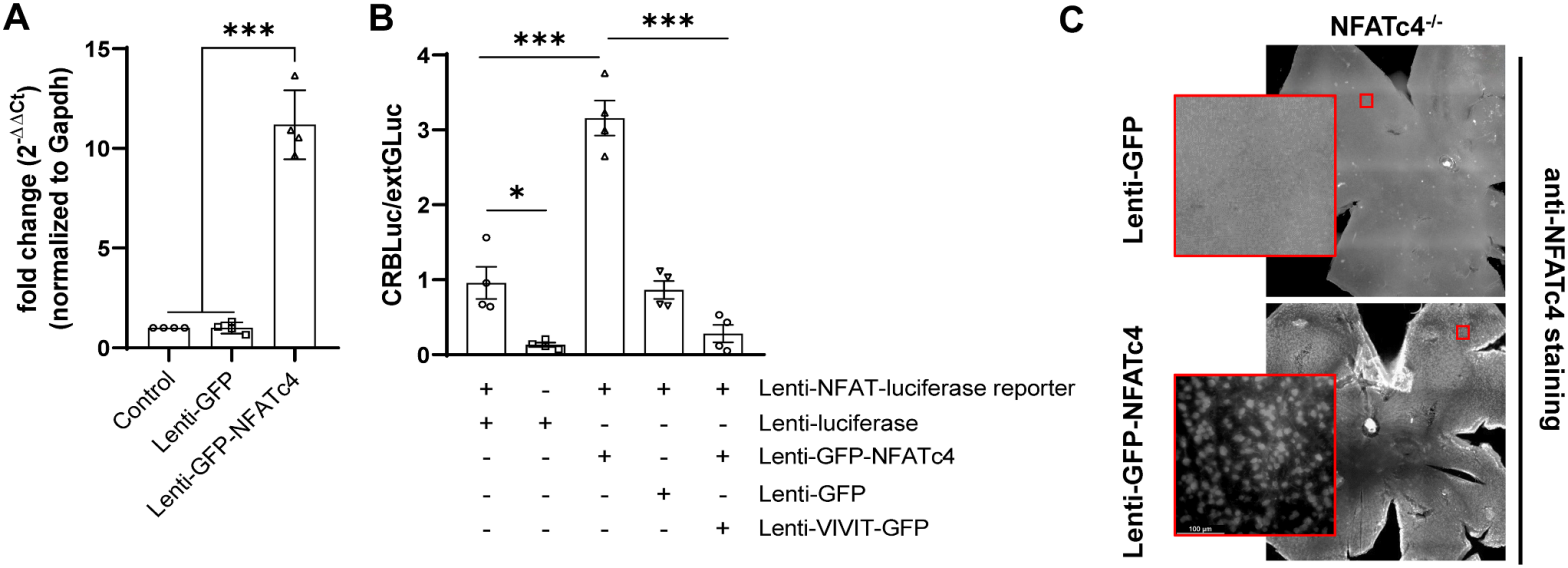
NFATc4 expression and NFAT transcriptional activity following lentiviral transduction. (**A**) Nfatc4 mRNA expression was assessed in primary hippocampal neurons following transduction with either Lenti-GFP-NFATc4 or Lenti-GFP, using real-time PCR. Raw data were normalized to Gapdh endogenous expression and calculated using 2^−ΔΔCt^ method. Nfatc4 expression level in non-transduced cells was set as 1. The data are presented as means ± SEM, with individual values obtained from n = 4 replicate treatment. (**B**) Primary hippocampal neurons were co-transduced with NFAT dual-reporter lentivirus and Lenti-GFP-NFATc4 (or other viruses as indicated on the graph) on DIV0 and cultured until DIV3. NFAT transcriptional activity was determined in cell lysates by measuring luciferase activity (n = 4). The results are expressed as a fold induction above baseline activity. The data are presented as means ± SEM, with individual values indicated on the graphs. * P < 0.05, *** P < 0.001. (**C**) Lenti-GFP or Lenti-GFP-NFATc4 were intravitreally injected into NFATc4^−/−^ retinas, followed by NFATc4 staining three weeks later. The retinas were stained using the antibodies indicated in *Retina cryosection staining* (primary: NFATc4, 1:500, Merck, USA; secondary: anti-rabbit conjugated to Alexa Fluor 488, 1:500, Thermo Fisher, USA). Representative images are presented. Scale bar: 100 μm

First, we tested the lentiviruses in vitro using primary hippocampal neurons. We observed notable viral efficiency, evidenced by an approximately 10-fold increase in NFATc4 mRNA level in GFP-NFATc4 overexpressing cells, as compared to Lenti-GFP transduced controls (Fig. 3A). Additionally, utilizing a lentiviral NFAT reporter, we confirmed that Lenti-GFP-NFATc4 overexpression was linked to higher baseline NFAT transcriptional activity (Fig. 3B). When Lenti-GFP-NFATc4 was intravitreally injected, NFATc4^−/−^ retinas exhibited significant NFATc4 expression three weeks post-transduction, contrasting with control retinas transduced with Lenti-GFP (Fig. 3C). These experiments highlight the effectiveness of Lenti-GFP-NFATc4 lentiviral injections to stably express functional NFATc4 in various systems, enabling rescue experiments in NFATc4 knockout mice.

**Fig. 4:**
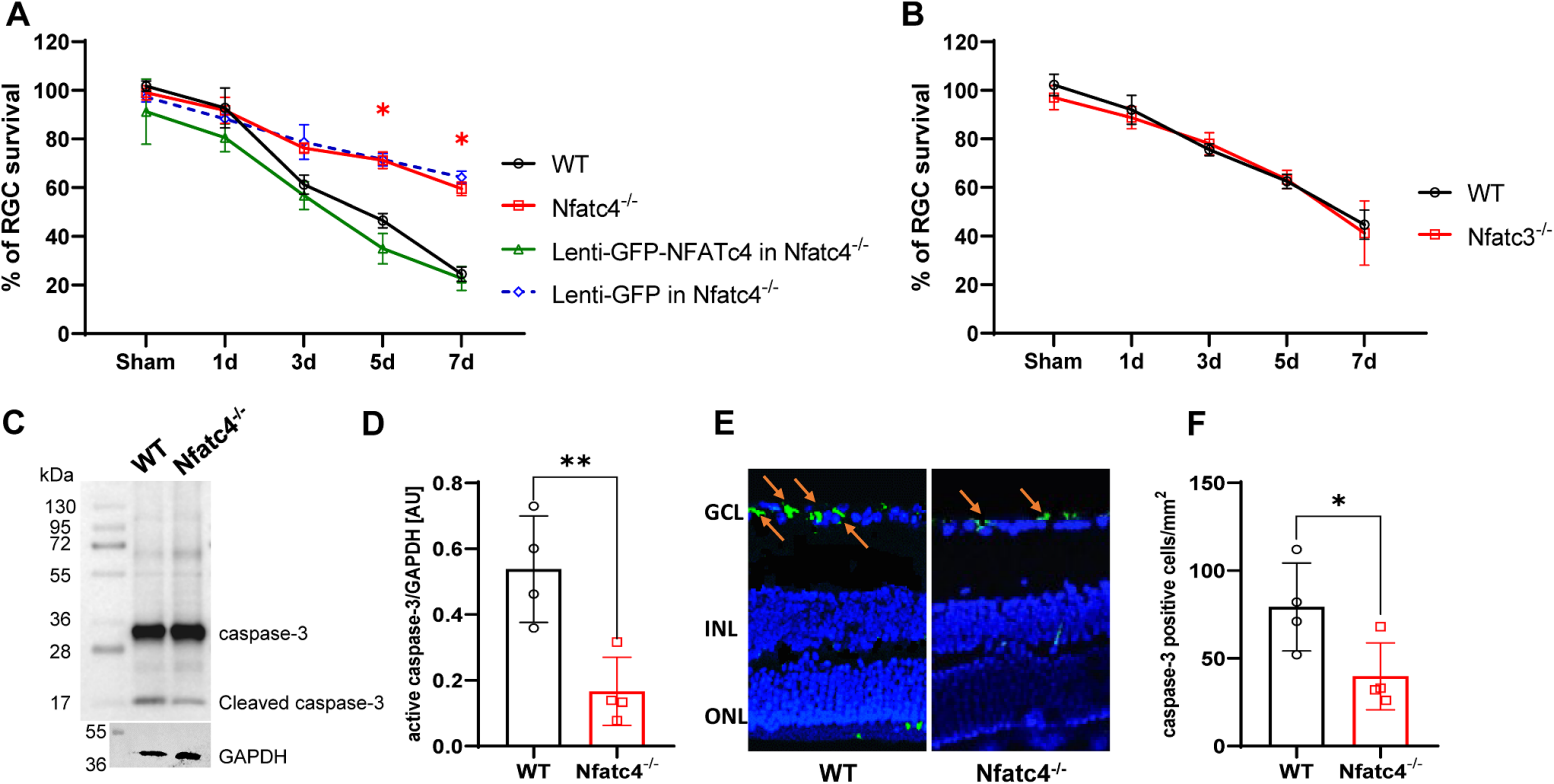
**Nfatc4**^**−/−**^ mice exhibit increased RGC survival after optic nerve injury. (**A**) The time course of RGC death after optic nerve injury in WT or Nfatc4^−/−^ mice with or without intravitreal injection with Lenti-GFP-NFATc4 or Lenti-GFP control virus. All points are n = 4 animals per point, normalized to naïve WT eyes. (**B**) The time course of RGC death after ONC showing no difference between WT and Nfatc3^−/−^ group, n = 4 animals per time point. RGC survival was normalized to WT naïve eyes. (**C**) Western blot analysis of caspase-3 and cleaved caspase-3 in a whole retina isolated on day 5 after ONC. GAPDH was used as a loading control. Representative blots are shown. (D) Quantification of cleaved caspase-3 protein level in WT or Nfatc4^−/−^ retinas following normalization to endogenous GAPDH level, n = 4. AU – arbitrary units. (**E**) Representative micrographs showing active caspase-3 staining in retina cryosections done on day 5 following ONC. The arrows indicate puncta corresponding to cleaved caspase-3 located in the ganglion cell layer (GCL). (**F**) Bar charts showing the quantitative analyses of average cleaved caspase-3–positive cell counts in the retina (n = 4, two images per sample). The data on the graph are presented as means ± SEM. * P < 0.05, ** P < 0.01

To assess the impact of NFATc4 knockout on RGC survival after injury, retinal flat mounts from adult NFATc4^−/−^ and wild-type controls were stained with RBPMS, and the labelled RGCs were quantified. The average number of RGCs in the NFATc4^−/−^ group (3159 ± 66 cells/mm^2^) did not show a significant difference from the wild-type group (3243 ± 103 cells/mm^2^), suggesting that NFATc4 is not essential for the generation of the normal RGC count (not shown). The enhancement in the survival of RBPMS-labeled RGCs became evident on day 5 following optic nerve crush in Nfatc4^−/−^ mice compared to wild-type mice (Fig. 4A). The rescue of NFATc4 expression through intravitreal injections of Lenti-GFP-NFATc4 into Nfatc4^−/−^ mice abolished this effect, resulting in a reduction of the RGC survival rate to the level observed in the wild-type control. Notably, there were no changes in RGC survival after ONC in Nfatc3^−/−^ mice compared to the wild-type (Fig. 4B).

Due to the expression of active caspase-3 by RGCs following axotomy [23], we next immunodetected caspase-3 and its cleaved form in whole retinas at 5 days after ONC. In NFATc4^−/−^ retinas, there was a significantly lower level of cleaved caspase-3 compared to the wild-type group (Fig. 4C-D). Immunocytochemical staining of retinal cryosections on day 5 (Fig. 4E) following the crush revealed a visibly lower signal for cleaved caspase-3 in the ganglion cell layer of NFATc4^−/−^ mice compared to the wild-type. As illustrated in Fig. 4F, the density profiles reflecting the immunoreactivity of caspase-3 were significantly decreased in the Nfact4^−/−^ group compared to the wild-type control. These findings suggest that the neuroprotective effect of Nfatc4 knockout is exerted, at least partly, through the modulation of apoptosis-related factors.

### NFATc4 Regulates Apoptotic Signaling in the Injured Retina

**Fig. 5:**
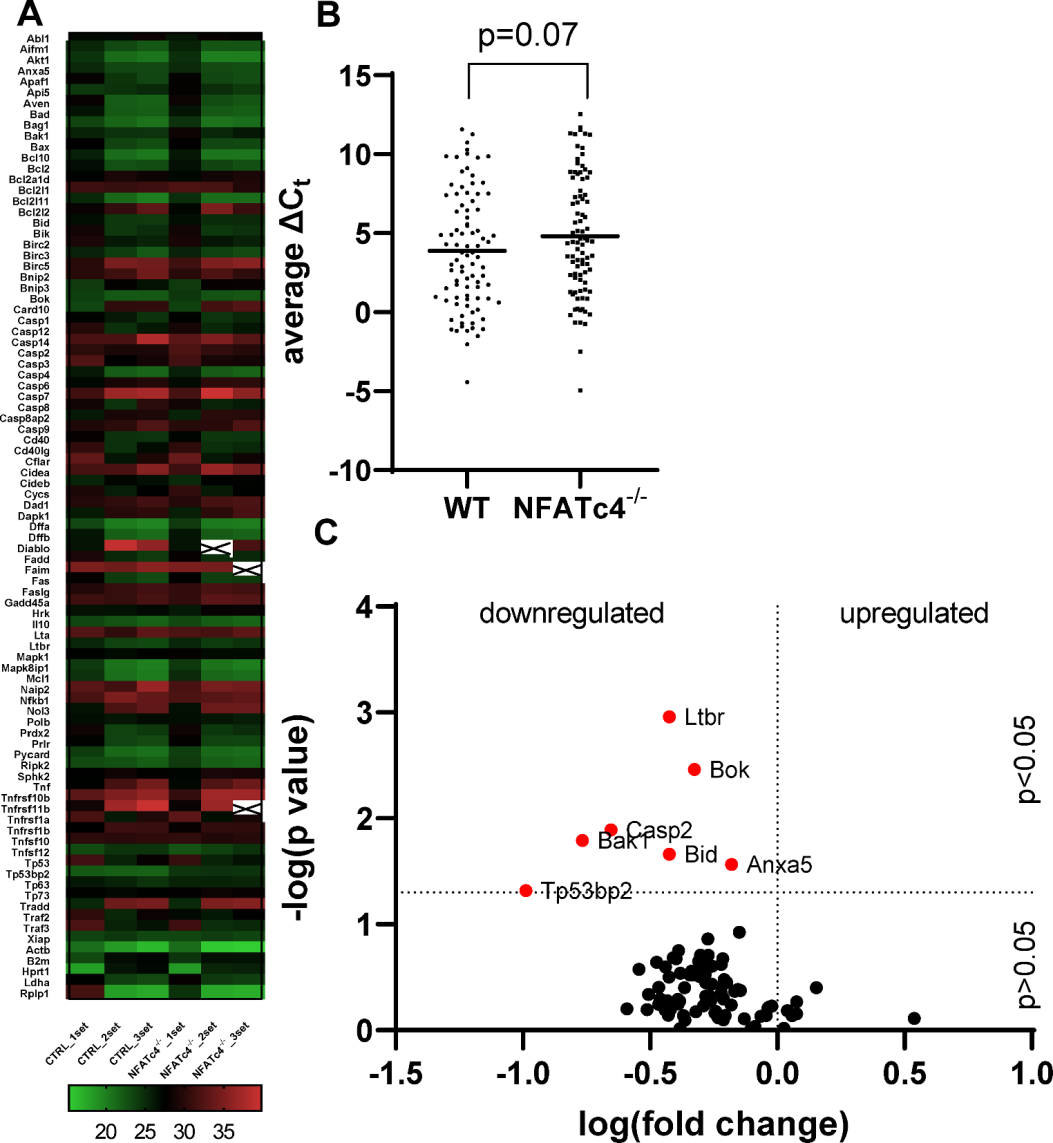
NFATc4 knockout downregulates pro-apoptotic signaling pathways 5 days after ONC.(**A**) Heat map adjusted to reflect variations in individual genes for each run. The map was generated using GraphPad Prism based on the microarray data. (**B**) Dot plot comparison of average C_t_ values of screened genes. The average is presented as a black horizontal line. (**C**) The volcano plot showing whole retina gene expression in WT and NFATc4^-/-^ mice following ONC. Genes (NFATc4^-/-^ vs. WT) with P<0.05 and fold change less than -2 are highlighted in red. Indicates undetected gene expression. Indicates undetected gene expression

As the activation of caspase-3 significantly decreased in NFATc4^−/−^ mice after ONC, we hypothesized that signaling pathways associated with apoptosis might be downregulated, thereby delaying RGC death. To investigate this, we conducted a microarray screening (the full list of genes can be found in Table 1) and observed a trend toward an increase in the average C_t_ values in the NFATc4^−/−^ group compared to the WT control, although the significance did not reach the 0.05 threshold (Fig. 5A-B). However, this trend could indicate a global decrease in apoptotic gene expression in NFATc4 knockout mice subjected to ONC.

**Table 1:** The list of screened apoptotic genes. The experiment was performed in triplicate and the data were analyzed using SABiosciences PCR Array Data Analysis Web Portal as described in *Materials and Methods* section

Gene bank	Gene symbol	Description
NM_001100850	Abl1	C-abl oncogene 1, receptor tyrosine kinase
NM_031356	Aifm1	Apoptosis-inducing factor, mitochondrion-associated 1
NM_033230	Akt1	V-akt murine thymoma viral oncogene homolog 1
NM_013132	Anxa5	Annexin A5
NM_023979	Apaf1	Apoptotic peptidase activating factor 1
NM_001127379	Api5	Apoptosis inhibitor 5
NM_001107757	Aven	Apoptosis, caspase activation inhibitor
NM_022698	Bad	BCL2-associated agonist of cell death
NM_001106647	Bag1	BCL2-associated athanogene
NM_053812	Bak1	BCL2-antagonist/killer 1
NM_017059	Bax	Bcl2-associated X protein
NM_031328	Bcl10	B-cell CLL/lymphoma 10
NM_016993	Bcl2	B-cell CLL/lymphoma 2
NM_133416	Bcl2a1d	B-cell leukemia/lymphoma 2 related protein A1d
NM_031535	Bcl2l1	Bcl2-like 1
NM_022612	Bcl2l11	BCL2-like 11 (apoptosis facilitator)
NM_021850	Bcl2l2	Bcl2-like 2
NM_022684	Bid	BH3 interacting domain death agonist
NM_053704	Bik	BCL2-interacting killer (apoptosis-inducing)
NM_021752	Birc2	Baculoviral IAP repeat-containing 2
NM_023987	Birc3	Baculoviral IAP repeat-containing 3
NM_022274	Birc5	Baculoviral IAP repeat-containing 5
NM_001106835	Bnip2	BCL2/adenovirus E1B interacting protein 2
NM_053420	Bnip3	BCL2/adenovirus E1B interacting protein 3
NM_017312	Bok	BCL2-related ovarian killer
NM_001130554	Card10	Caspase recruitment domain family, member 10
NM_012762	Casp1	Caspase 1
NM_130422	Casp12	Caspase 12
XM_234878	Casp14	Caspase 14
NM_022522	Casp2	Caspase 2
NM_012922	Casp3	Caspase 3
NM_053736	Casp4	Caspase 4, apoptosis-related cysteine peptidase
NM_031775	Casp6	Caspase 6
NM_022260	Casp7	Caspase 7
NM_022277	Casp8	Caspase 8
NM_001107921	Casp8ap2	Caspase 8 associated protein 2
NM_031632	Casp9	Caspase 9, apoptosis-related cysteine peptidase
NM_134360	Cd40	CD40 molecule, TNF receptor superfamily member 5
NM_053353	Cd40lg	CD40 ligand
NM_057138	Cflar	CASP8 and FADD-like apoptosis regulator
NM_001170467	Cidea	Cell death-inducing DFFA-like effector a
NM_001108869	Cideb	Cell death-inducing DFFA-like effector b
NM_012839	Cycs	Cytochrome c, somatic
NM_138910	Dad1	Defender against cell death 1
NM_001107335	Dapk1	Death associated protein kinase 1
NM_053679	Dffa	DNA fragmentation factor, alpha subunit
NM_053362	Dffb	DNA fragmentation factor, beta polypeptide (caspase-activated DNase)
NM_001008292	Diablo	Diablo homolog (Drosophila)
NM_152937	Fadd	Fas (TNFRSF6)-associated via death domain
NM_080895	Faim	Fas apoptotic inhibitory molecule
NM_139194	Fas	Fas (TNF receptor superfamily, member 6)
NM_012908	Faslg	Fas ligand (TNF superfamily, member 6)
NM_024127	Gadd45a	Growth arrest and DNA-damage-inducible, alpha
NM_057130	Hrk	Harakiri, BCL2 interacting protein (contains only BH3 domain)
NM_012854	Il10	Interleukin 10
NM_080769	Lta	Lymphotoxin alpha (TNF superfamily, member 1)
NM_053842	Mapk1	Mitogen activated protein kinase 1
NM_053777	Mapk8ip1	Mitogen-activated protein kinase 8 interacting protein 1
NM_021846	Mcl1	Myeloid cell leukemia sequence 1
XM_226742	Naip2	NLR family, apoptosis inhibitory protein 2
XM_342346	Nfkb1	Nuclear factor of kappa light polypeptide gene enhancer in B-cells 1
NM_053516	Nol3	Nucleolar protein 3 (apoptosis repressor with CARD domain)
NM_017141	Polb	Polymerase (DNA directed), beta
NM_017169	Prdx2	Peroxiredoxin 2
NM_012630	Prlr	Prolactin receptor
NM_172322	Pycard	PYD and CARD domain containing
XM_342810	Ripk2	Receptor-interacting serine-threonine kinase 2
NM_001012066	Sphk2	Sphingosine kinase 2
NM_012675	Tnf	Tumor necrosis factor (TNF superfamily, member 2)
NM_001108873	Tnfrsf10b	Tumor necrosis factor receptor superfamily, member 10b
NM_012870	Tnfrsf11b	Tumor necrosis factor receptor superfamily, member 11b
NM_013091	Tnfrsf1a	Tumor necrosis factor receptor superfamily, member 1a
NM_130426	Tnfrsf1b	Tumor necrosis factor receptor superfamily, member 1b
NM_145681	Tnfsf10	Tumor necrosis factor (ligand) superfamily, member 10
NM_001001513	Tnfsf12	Tumor necrosis factor ligand superfamily member 12
NM_030989	Tp53	Tumor protein p53
XM_223012	Tp53bp2	Tumor protein p53 binding protein, 2
NM_019221	Tp63	Tumor protein p63
NM_001108696	Tp73	Tumor protein p73
NM_001100480	Tradd	TNFRSF1A-associated via death domain
NM_001107815	Traf2	Tnf receptor-associated factor 2
NM_001108724	Traf3	Tnf receptor-associated factor 3
NM_022231	Xiap	X-linked inhibitor of apoptosis

Considering a 2-fold change as a minimum and P < 0.05, we identified 7 genes with significantly downregulated expression: *Ltbr*, *Bok*, *Casp2*, *Bak1*, *Bid*, *Anxa5*, *Tp53bp2* (Fig. 5C). These genes include components of the tumor necrosis factor receptor superfamily, Bcl-2 protein family members, caspase superfamily, and apoptosis-stimulating protein of p53 family. Generally, the function of proteins encoded by these genes is considered pro-apoptotic. Therefore, the downregulation of apoptosis-promoting genes in response to NFATc4^−/−^ knockout may restrict ONC-induced RGC death.

### NFATc4 Knockout Improves Retina Function Following Optic Nerve Crush

**Fig. 6:**
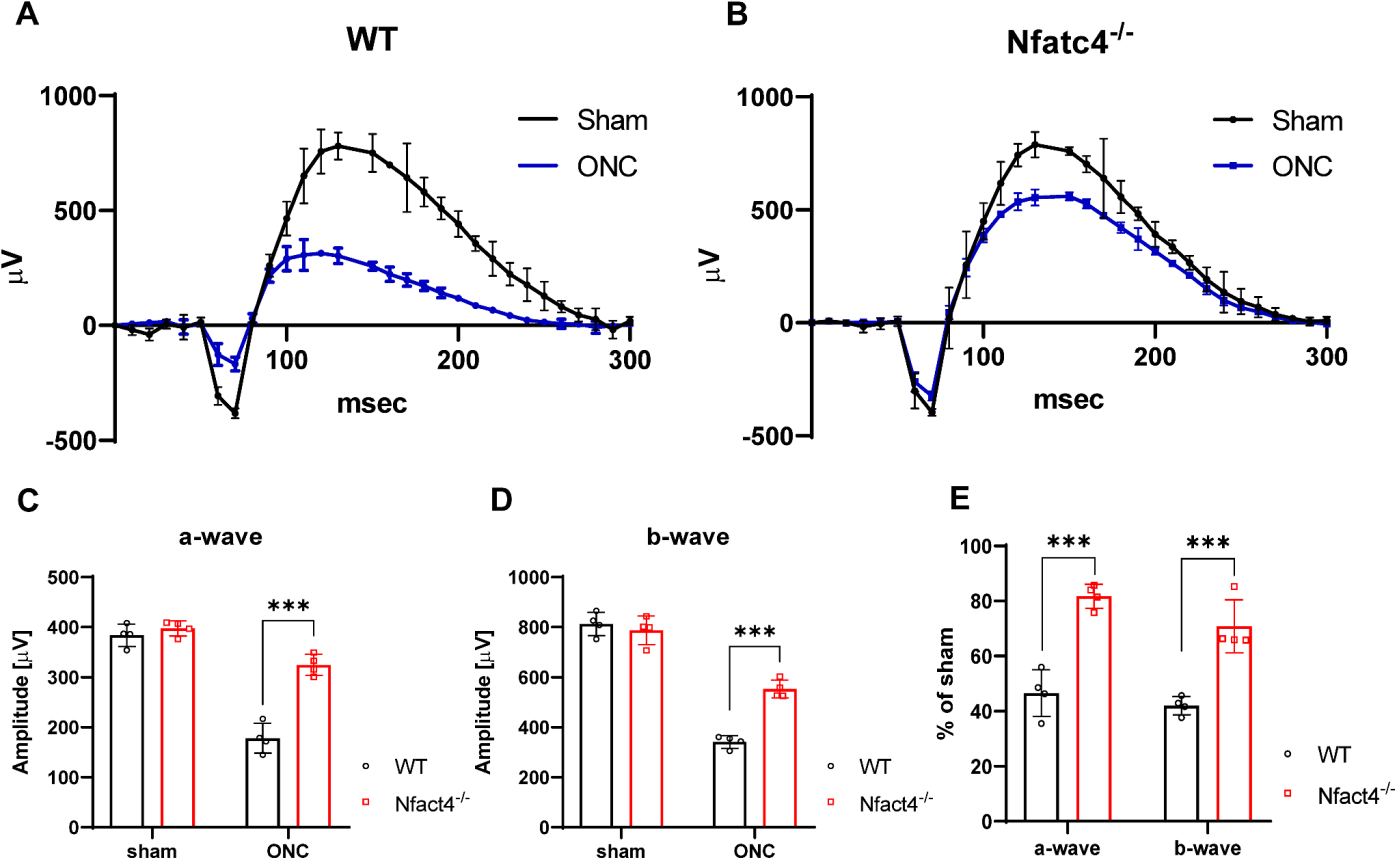
Effect of NFATc4 knockout on electroretinogram responses. (**A**) Representative ERG traces for wild-type mice recorded before and 5 days after ONC. (**B**) Representative ERG traces for Nfatc4^−/−^ mice recorded before and 5 days after ONC. Each ERG was obtained by averaging two responses to 2.48 cd-s/m^2^ flashes with an interstimulus interval of 2 minutes. (**C**) Analysis of ERG a-wave amplitudes in WT and Nfatc4^−/−^ mice before and 5 days after ONC. (**D**) Analysis of ERG b-wave amplitudes in WT and Nfatc4^−/−^ mice before and 5 days after ONC. (**E**) Normalized a-wave and b-wave amplitudes 5 days after ONC. The data on the graph are presented as means ± SEM. *** P < 0.001, n = 4

To investigate whether the enhanced RGC survival in Nfatc4^−/−^ mice correlated with the retina’s response to flash stimuli, we utilized scotopic ERG (Fig. 6A-E). In wild-type mice, the average baseline amplitudes of a- and b-waves were 383.7 ± 9.6 µV and 813 ± 20.3 µV, respectively. In Nfatc4^−/−^ mice, the average baseline amplitudes of a- and b-waves were 397.2 ± 6.5 µV and 787.5 ± 24.8 µV, respectively. In wild type mice, a- and b-waves recorded 5 days post-crush were decreased by 53.5 ± 7.2% and 58 ± 3.1%, respectively, when compared to the sham control. ERG performed in Nfatc4^−/−^ mice on the same post-crush day showed a reduction in a- and b-waves by 18.2 ± 2.7% and 29.6 ± 2.8%, respectively. Although the ERG waves were also reduced in Nfatc4^−/−^ mice when compared to the sham group, these deficits were significantly less than in wild-type mice, demonstrating the positive effect of NFATc4 knockout on the function of the injured retina.

### NFATc4 Controls Axon Regeneration After Optic Nerve Injury

**Fig. 7:**
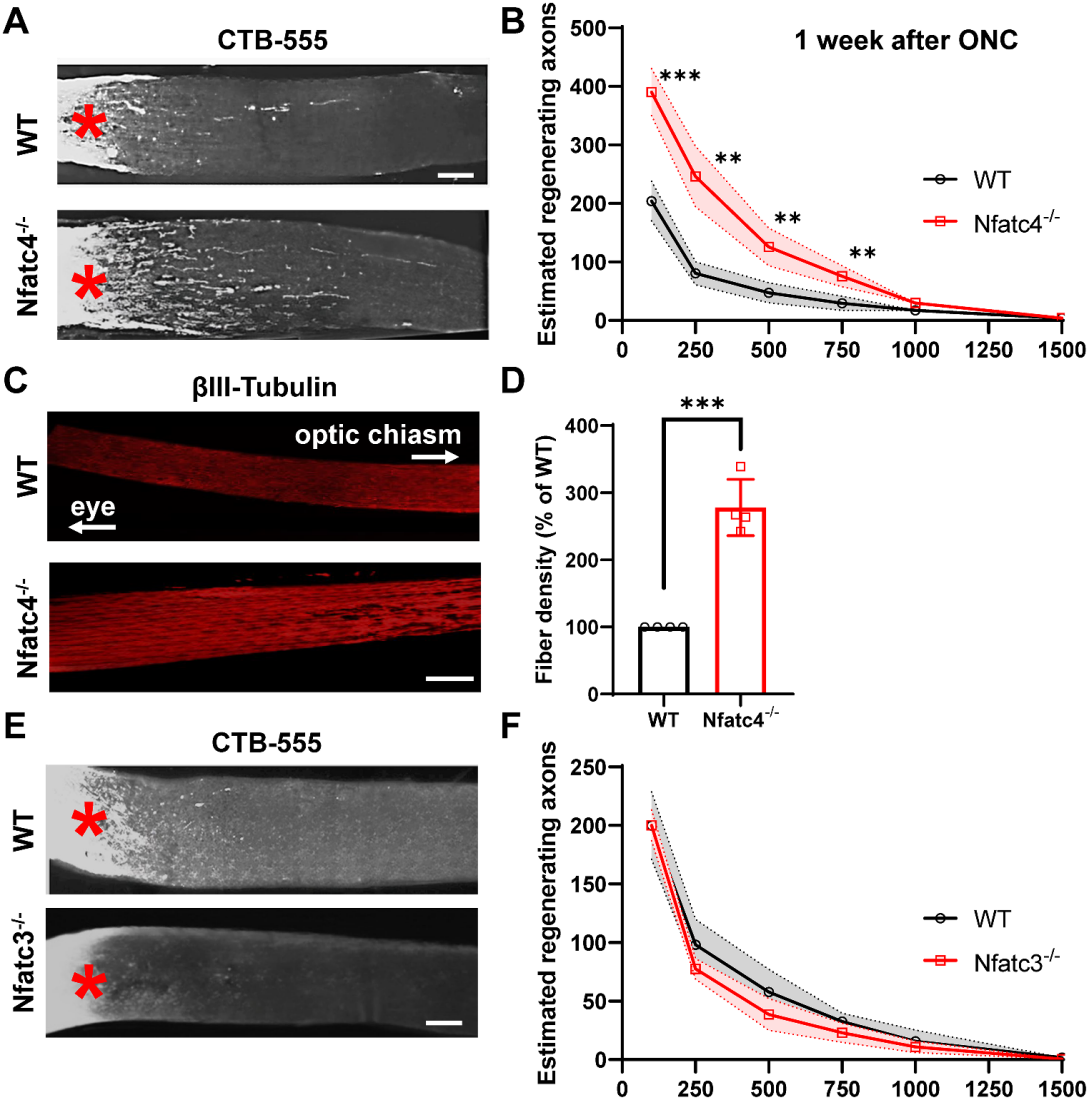
Effect of NFATc4 knockout on axon regeneration after optic nerve crush (**A**) Representative images showing CTB-labelled regenerating axons in NFATc4^−/−^ mice on day 7 after optic nerve crush. (**B**) Quantification of regenerating axons from the injury site, n = 6 per group. (**C**) βIII-tubulin staining showing delayed optic nerve degeneration in NFATc4^−/−^ mice. (**D**) Quantification of fiber density, n = 4. (**E**) Representative images of regenerating axons in NFATc3^−/−^ mice on day 7 post-crush. (**F**) Quantification of regenerating axons, n = 6 per group. Asterisks mark the crush site. The data on the graph are presented as means ± SEM. ** P < 0.01, *** P < 0.001. Scale bar: 250 μm

Next, we investigated the effect of NFATc4 knockout on short-term axon regeneration. To address this question, RGCs were labelled by intravitreal injection of cholera toxin-B subunit (CTB) and the number of axons was quantified at day 7 after ONC. The number of regenerating axons that extended 100, 250, 500, 750 μm beyond the crush point was significantly higher in the NFATc4^−/−^ group than in the wild type (Fig. 7A-B). We also measured βIII-tubulin expression within the axons to determine axonal integrity. The density of βIII-tubulin assessed 500 μm from the crush point was significantly higher in the NFATc4^−/−^ group compared to the wild type (Fig. 7C-D), suggesting delayed axonal degeneration. Interestingly, no enhanced regeneration after ONC was seen in NFATc3^−/−^ mice (Fig. 7E-F). Taken together, our results suggest that NFATc4 may promote RGC death and repress regeneration of the injured optic nerve.

## Discussion

The research on NFAT transcription factors in RGC survival and regeneration following injury has been largely discontinued since 2014 when Xu and colleagues demonstrated an overlapping pattern of NFATc4, cleaved caspase-3, and FasL in a light-induced model of retinal degeneration [36]. Using purified RGCs, as well as Nfatc4^−/−^ or NFATc3^−/−^ knockout mice and lentiviral-mediated gene delivery, we demonstrate that NFATc4 plays a crucial role in RGC survival in a model of optic nerve crush. The knockout of NFATc4 significantly improved RGC function and enhanced axonal regeneration in the injured retina. The critical role of NFATc4 is highlighted by the fact that no similar changes were observed in Nfatc3^−/−^ knockout mice. This suggests that NFATc4 in the retina is downstream of divergent signaling pathways mediating survival and regeneration in the presence or absence of neurotrophic factors.

NFATc4 belongs to the family of Rel homology domain (RHR) and NFAT homology domain (NHR)-containing transcription factors (NFATc1-c4), whose activity is controlled in a Ca^2+^- and CaN-dependent manner. The NHR contains two CaN-binding motifs: a Ca^2+^ - independent PXIXIT motif in the N terminus and a Ca^2+^ -dependent LxVP motif in the C-terminal portion of NHR [64]. Despite shared activation by CaN-dependent dephosphorylation, the activity of specific NFAT isoforms within distinct populations of neuronal cells is controlled through poorly understood mechanisms. For example, NFATc4’s activity was selectively required for the survival of adult-born neurons in response to BDNF [65] and mediated anti-apoptotic transcription in NMDA receptor-stimulated cortical neurons [66]. Depending on its transcriptional activity, NFATc4 may also participate in pro-apoptotic signaling, usually combined with an extrinsic pathway-dependent increase in Fas ligand (FasL) expression. Gomez-Sintes and Lucas demonstrated that increased nuclear NFATc4 translocation correlated with elevated FasL levels and Fas activation, an effect absent in Fas-deficient *Ipr* mice and following cyclosporine administration [67]. Similarly, NFATc4-mediated FasL up-regulation has been proposed to underlie methamphetamine-induced neuronal loss [68]. Furthermore, deafferentiation-induced neuronal apoptosis in the cochlear nucleus has also been suggested to be mediated by NFATc4/FasL activation [69]. Hence, the opposite functions played by NFATc4 may be attributed to the upstream stimulus controlling its phosphorylation/dephosphorylation ratio or be cell-specific, as different cells can selectively activate specific NFAT isoforms depending on environmental cues [70–75].

The role of NFATs in RGCs is still not fully understood. Our results demonstrate that NFATc4 is specifically and transiently up-regulated in the retina after optic nerve injury. The time-course of NFATc4 increase in our experimental model is similar to the one observed in [36], suggesting a more general phenomenon. Another research group has also demonstrated a change in NFATc4 expression in response to optic nerve injury. The microarray hybridization screen performed by Lukas and colleagues within 6 h post injury revealed early downregulation of NFATc4 in the ganglion cell layer [76]. This observation was confirmed by a more recent analysis of the retinal transcriptome performed at the same time point after ONC [77]. Both studies clearly demonstrate changes in NFATc4 expression; however, they focus either on changes occurring early after ONC or performed the injury in embryonic (E20) and postnatal animals (P1-P3). Moreover, there were significant differences between postnatal and embryonic NFATc4 expression. It is known that capacity of RGC for axonal growth and the regeneration of injured axons sharply decreases soon after birth, and this age-dependent decline is associated with a profound reorganization of retinal transcriptome [43, 78]. A growing body of evidence indicates that molecular changes in the injured retina are progressive and many of them appear later in time [76, 79–81]. Therefore, it is not unexpected that the NFATc4 expression profile changes over time as RGC death becomes prominent. Consistent with our study, none of the transcriptional profiling analyses revealed changes in other NFAT isoforms after ONC.

Based on our data, wherein NFATc4 knockdown promotes RGC survival in vivo, and lentiviral-mediated NFATc4 expression in Nfatc4^−/−^ mouse reverses this pro-survival effect, the up-regulation of NFATc4 following injury likely represents an attempted pro-apoptotic response. This NFATc4-mediated response seems to be specifically induced by the injury, as the number of RGCs in uninjured wild type and Nfatc4^−/−^ groups was unchanged and similar to the results previously reported for the C57BL/6 mouse [82]. This would indicate that NFATc4 expression is dispensible for normal retina development or in uninjured RGCs. The importance of CaN/NFAT signaling in retinal degeneration has been suggested by several groups. Freeman and Grosskreutz demonstrated that the administration of the FKBP12 ligand FK506 increased the number of RGCs following optic nerve crush [83]. The FK506-FKBP12 complex is expected to inhibit CaN phosphatase activity and decrease NFAT dephosphorylation, thus preventing its nuclear import. Moreover, it has been demonstrated that CaN is activated in response to ocular hypertension in the mouse model of glaucoma [84] and is responsible for RGC degeneration [85]. In view of that, knockdown of NFATc4 in vivo may disrupt calcineurin/NFATc4 downstream signaling and, at least in part, attenuate massive apoptosis of injured RGCs. This posits NFATc4 as one of the important mediators of RGC death following optic nerve crush.

While the data suggests NFATc4’s involvement in RGC death, the relevance of NFATc4 function as a potential target for axonal regeneration after retina injury has not been previously explored. Using CTB and βIII-tubulin staining, we demonstrated that NFATc4 knockout delayed axon degeneration. Labeling axons with CTB is a reliable technique based on axonal transport that is widely used for monitoring axonal regeneration [41]. However, around day 7 post-crush, axonal transport is significantly altered, leading to distal axon terminal degeneration [86]. Because, in our experiment, CTB was injected 2 days before retina collection, it is also plausible that NFATc4 knockout may affect dye transport, eventually influencing the labelling pattern one week after ONC. Nonetheless, visualization of remaining axons with βIII-tubulin, which is a marker of axonal integrity [52], seems to confirm that NFATc4 plays a role in delaying axonal disintegration one week after ONC.

It is hypothesized that axonal transport breakdown is preceded by a lesion-induced signaling, triggering axon swelling and irreversible changes in neurofilaments and microtubules integrity [87–89]. In their elegant set of experiments, Knöferle and colleagues linked axotomy-induced intraaxonal Ca^2+^ elevation to a secondary generation of autophagosomes that participate in axonal degradation [90]. The initial increase in Ca^2+^ concentration activating CaN is an obligatory step for the activation of NFAT-dependent transcription. Moreover, recent reports suggest an important contribution of NFAT to autophagy in retinal pigmental epithelial cells [91] as well as in other cell types [92]. In view of this, it is tempting to speculate that Ca^2+^-dependent activation of NFATc4 and NFATc4 downstream signaling should be placed among important events restricting axonal regeneration after mechanical injury.

The remaining question is how NFATc4 knockout slows down the time-dependent apoptosis of injured RGC. NFAT proteins can directly regulate the expression of apoptosis-related genes along with the induction of pro-inflammatory cytokine production [93–98]. Both apoptosis and neuroinflammation are frequently associated with multiple neurodegenerative diseases [99–101]. Although it would be interesting to explore whether the modulation of retinal inflammation underlies enhanced RGC survival in NFATc4^−/−^ mouse, our observation of lowered caspase-3 cleavage directed us toward studying apoptosis-related genes. The microarray analysis revealed that certain pro-apoptotic genes are downregulated in NFATc4^−/−^ mice, indicating that increased RGC survival observed in this group after ONC may arise from blocking the apoptotic program. This is consistent with a prior study showing reduced sensitivity of sensory hair cells to TNF-mediated apoptosis in NFATc4^−/−^ mice [102]. In addition, Bak1, Bok, and Bid, part of the Bcl-2 family of apoptosis regulators, were downregulated in the NFATc4^−/−^ retina after ONC. Selective repression of BAK1 protein attenuated neuronal apoptosis [103], similar to Bax/Bak1 double knockout cells that are resistant to multiple apoptotic inducers [104, 105]. Like BAK1 and BAX, BOK is a pro-apoptotic protein that can induce mitochondrial apoptosis [106]. In line with this finding, Bok^−/−^ cells were partially protected from ER stress-induced apoptosis elicited by thapsigargin or bortezomib [107]. On the contrary, other studies suggested a lack of its role in apoptosis as Bok knockout does not alter responsiveness to various apoptotic stimuli [108, 109]. Similarly, Bid-deficient mice are resistant to Fas-induced apoptosis [110], and Tp53bp2 downregulation protected from apoptosis in certain cell types [111, 112]. However, which NFATc4-dependent changes in gene expression reflect a pro-survival response, improving RGC survival and delaying axonal degeneration, needs further attention. It has been recently demonstrated that among 46 different RGC subtypes distinguished by high-throughput single-cell RNA-seq [113],some types exhibit selective resilience to injury while others are more susceptible to degeneration and die quickly [114]. Since NFATc4 may affect the expression profile of genes involved in apoptosis, certain types of RGCs may be more vulnerable because of their NFATc4 expression, consistent with our data that NFATc4 up-regulation peaked 1 day after ONC.

NFATc4 is unique among other NFAT isoforms in its regulation by upstream signaling in neurons. Unlike NFATc3, activation of NFATc4 requires a coincident elevation in intracellular Ca^2+^ and suppression of glycogen synthase kinase 3β (GSK-3β) [74]. GSK-3β and other kinases are known to phosphorylate multiple serines in the NFAT regulatory domain, leading to the termination of NFAT-dependent gene expression [115–117]. It has not been fully resolved whether the activity of phosphorylating/dephosphorylating enzymes is an organized mechanism. In view of this, an interesting question that remains elusive is how the activity of NFATc4 is orchestrated to direct the RGC response to injury and affect the regeneration of injured axons. Our previous study [47] demonstrated that manipulation within A-kinase anchoring protein 6 (AKAP6)-organized pro-survival signaling significantly enhanced RGC survival following ONC. AKAP6 brings together calcineurin [118], ERK5 [119] and NFAT transcription factor (unpublished data), providing a platform for the integration of pro-survival an pro-death signaling. Depending on the upstream stimuli, ERK5 activity can be effectively counterbalanced by locally anchored CaN, with the relevant outcome toward NFATc4 downstream signaling. Up to now, more than fifty AKAPs have been identified that are involved in different cellular processes. This abundance allows for efficient spatial and temporal control of intracellular signaling, but which AKAPs may potentially participate in RGC survival requires further investigation.

It has been demonstrated that distinct NFAT isoforms may antagonize each other in the control of gene expression in retina degeneration [120]. For instance, siRNA-mediated NFATc3 knockdown increased the expression of TNFα-induced inflammatory response, whereas downregulation of NFATc4 has the opposite effects. Several molecular therapies based on pharmacological NFAT inhibition have been described to carry substantial potential toward retina degeneration [35, 121]. It is highly likely that greater efficacy could be achieved by identifying the NFAT isoform’s role in RGC degeneration, which would give rise to development of isoform-specific therapies. Therefore, our intent was to investigate how NFAT isoforms contribute to the pathological events underlying injury-mediated RGC loss. To our best knowledge, no similar study with NFATc4 or NFATc3 knockout animals has been performed up to now.

In summary, our data suggest that NFATc4 should be considered one of the major regulators of adult RGC survival following injury, and central to the complex interplay of multiple molecular events in axonal regeneration. Further studies on NFATc4 and, in particular, the co-regulators of its transcriptional activity are essential, as they may lead to new therapeutic interventions allowing for the preservation of RGC function. Despite accumulating studies on gene therapy enhancing RGC survival and axon regeneration, the search for novel target molecules is of paramount importance, as the functional restoration of visual pathways still remains a challenge. The synergistic effect of NFATc4 downregulation along with other known axon regeneration promoters may provide an effective combinatorial strategy to improve vision impairments in optic neuropathies.

## Electronic Supplementary Material

Below is the link to the electronic supplementary material.

Supplementary Material 1

## Data Availability

The datasets used and/or analyzed during the study are available upon requests from the corresponding authors.
