# Supplementary figures and images for: NFATc4 Knockout Promotes Neuroprotection and Retinal Ganglion Cell Regeneration After Optic Nerve Injury
Source: Mol Neurobiol. 2024 Apr 19;61(11):9383–401. doi: 10.1007/s12035-024-04129-0 (PMC11496353; doi:10.1007/s12035-024-04129-0)

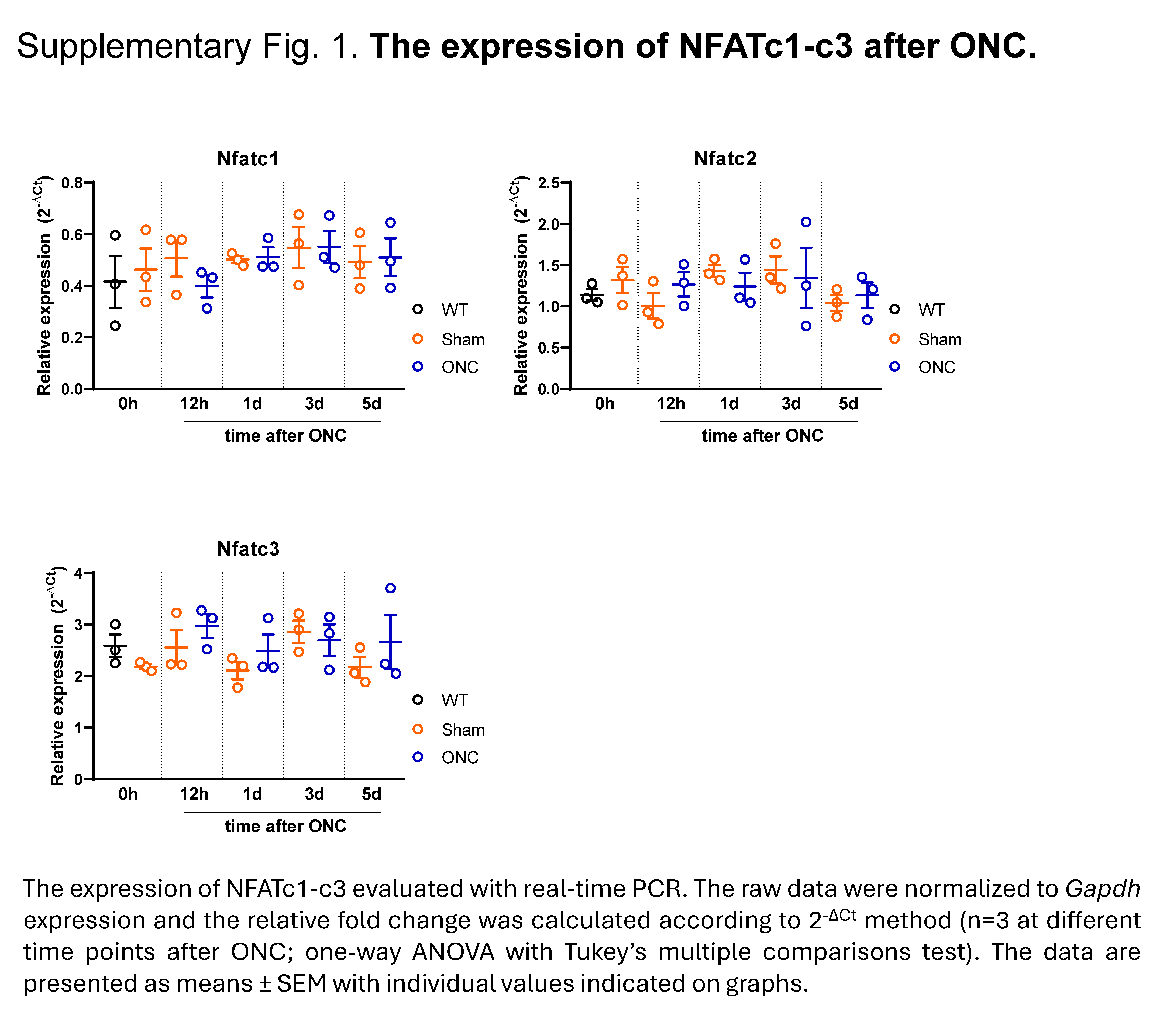

Supplement: Supplementary file 1 — Supplementary Material 1 [file 12035_2024_4129_MOESM1_ESM.tif]
